# Roles of microRNA on cancer cell metabolism

**DOI:** 10.1186/1479-5876-10-228

**Published:** 2012-11-20

**Authors:** Bing Chen, Hongbin Li, Xiao Zeng, Pengbo Yang, Xinyu Liu, Xia Zhao, Shufang Liang

**Affiliations:** 1 State Key Laboratory of Biotherapy, West China Hospital, Sichuan University, No.17, third section of Renmin South Road, Chengdu 610041, People’s Republic China; 2 Department of Gynecology and Obstetrics, West China Second Hospital, Sichuan University, Chengdu, 610041, People’s Republic China

**Keywords:** MicroRNA, Cell metabolism, MiRNA biomarker

## Abstract

Advanced studies of microRNAs (miRNAs) have revealed their manifold biological functions, including control of cell proliferation, cell cycle and cell death. However, it seems that their roles as key regulators of metabolism have drawn more and more attention in the recent years. Cancer cells display increased metabolic autonomy in comparison to non-transformed cells, taking up nutrients and metabolizing them in pathways that support growth and proliferation. MiRNAs regulate cell metabolic processes through complicated mechanisms, including directly targeting key enzymes or transporters of metabolic processes and regulating transcription factors, oncogenes / tumor suppressors as well as multiple oncogenic signaling pathways. MiRNAs like miR-375, miR-143, miR-14 and miR-29b participate in controlling cancer cell metabolism by regulating the expression of genes whose protein products either directly regulate metabolic machinery or indirectly modulate the expression of metabolic enzymes, serving as master regulators, which will hopefully lead to a new therapeutic strategy for malignant cancer. This review focuses on miRNA regulations of cancer cell metabolism,including glucose uptake, glycolysis, tricarboxylic acid cycle and insulin production, lipid metabolism and amino acid biogenesis, as well as several oncogenic signaling pathways. Furthermore, the challenges of miRNA-based strategies for cancer diagnosis, prognosis and therapeutics have been discussed.

## Background

MicroRNAs (miRNAs) are endogenous ~22 nt RNAs that can play important regulatory roles in a variety of biological processes. They are genome-encoded, endogenous negative regulators of translation and mRNA stability originating from long primary transcripts with local hairpin structures [1]. Conserved seed pairing indicates that over one third of human genes appear to be conserved miRNA targets [2]. MiRNAs are involved in cell proliferation, intercellular signaling, cell growth, cell death [3,4], cellular differentiation, apoptosis [5] and cellular metabolism [6,7]. Meanwhile they have emerged as key post-transcriptional regulators of gene expression, and their dysregulation may lead to abnormal gene expression, which is associated to human diseases such as cancer. For example, miR-378* (expressed from the 3'-arm), which mediates metabolic shift in breast cancer cells, leading to a reduction in tricarboxylic acid cycle gene expression and oxygen consumption as well as an increase in lactate production, via the PGC-1β/ERRγ transcriptional pathway [8].

Recent studies have shown that miRNAs play important roles in energy metabolism, including glucose and lipid metabolism and amino acid biogenesis [9] (Table 1). Besides, miRNAs are also able to recognize and modulate metabolic factors in transcriptional levels, relevant both in non-neoplastic and in cancer cells [10]. The altered metabolism of tumor cells may be a potential means to evade programmed cell death in order to favor survival and growth. The best characterized metabolic phenotype observed in tumor cells is the Warburg effect, in which the deregulation of miRNAs contributes to high glycolysis [11,12].

**Table 1:** Summary of miRNA regulation in energy metabolism

**miRNA**	**Tissue / cell lines**	**miRNA functions**	**Target gene/Pathway**	**Reference**
miR-103/107	obese mice: ob/ob mice and diet-induced-obese (DIO) C57BL/6J mice	regulate insulin sensitivity	caveolin-1	41
miR-122	primary mouse hepatocytes and AML12	regulator of cholesterol and fatty-acid metabolism		23,26,38,49,5054,60
miR-133	293FT cells	decreased GLUT4 expression and reduced insulin-mediated glucose uptake in cardio myocytes		31,34
miR-14	*Drosophila*	regulate fat metabolism		36
miR-143	liver of obese mouse models	impairs insulin-stimulated AKT activation and glucose homeostasis	*Orp8* / *Akt* pathway	12,26,35,36, 37,91
miR-146	diabetic db/db mice islets / MIN6B1 cells	cell death	*Irak1* &*Traf6 */ AP-1 pathway	52
miR-15a/16-1	leukemic cell line model (MEG-01) and in primary CLL samples	directly or indirectly affect apoptosis and cell cycle	*MCL1, BCL2, ETS1,or JUN*	27
miR-195-5p	bladder cancer T24 cells	inhibited cell growth and promoted cell apoptosis through suppression of GLUT3 expression		25
miR-210	human pulmonary arterial endothelial cells (HPAECs)	cellular metabolism and adaptation to cellular stress	*ISCU1/2*	42
miR-23a/b	human P-493 B cells	regulate expression of glutaminase and glutamine metabolism	*c-Myc*	6
miR-277	*D. melanogaster*	a metabolic switch controlling amino acid catabolism		61
miR-27a	3T3-L1	suppress adipocyte differentiation	PPARγ	51
	Male C57BL/6J mice and 3T3-L1 cells	a negative regulator of adipocyte differentiation		
miR-29b	human kidney cells (HEK293)	control metabolic pathway of amino acid catabolism	mRNA for DBT	62
miR-335	liver of obese mouse	affects adipocyte differentiation and lipid accumulation	*PPAR*γ &*aP2*	53
miR-33a/b	mouse peritoneal macrophages	regulate both HDL biogenesis in the liver and cellular cholesterol efflux	ABCA1	58
miR-34a	diabetic db/db mice islets / MIN6B1 cells	sensitization to apoptosis and impaired nutrient-induced secretion	*BclII* / p53 pathway	52,68
miR-370	liver of mouse	affects lipid metabolism	*Cpt1*α	54
miR-375	pancreatic endocrine cells (MIN6 cells)	suppressed glucose-induced insulin secretion	*Mtpn*	45,76
miR-378	NMuMG cells and NT2196	reduce tricarboxylic acid cycle gene expression and oxygen consumption as well as increase lactate production	ERRγ and GABPA	8
	INS-1E cells/primary rat islets	decreased glucose-stimulatory action on insulin gene expression and DNA synthesis		
		Cell growth	*Eef1e1*	76
		cell growth	*Cadm1*	76
		negatively regulate cellular growth and proliferation	*C1qbp*	76
		regulate cell cycle and cellular proliferation	*Cav1* / p53 pathway or MAPK pathway	76,79
		angiogenesis and cell proliferation	*Id3* / VEGF pathway or MAPK pathway	76,79
		regulate cell growth/survival	*Rasd1 / Ras* pathway	76,81
		cell-cell signaling	*Rgs16*	76
		mitochondrial morphology and cristae structure, cell survival and death	*Aifm1*	76
		negative regulation of proliferative activity	*HuD*	76,83

MiRNAs participate in controlling cancer cell metabolism by regulating the expression of genes whose protein products either directly regulate metabolic machinery or indirectly modulate the expression of metabolic enzymes, serving as master regulators. Generally, miRNA signatures may distinguish physiological, pathologic from cancerous states, which could be useful biomarkers in targeted therapeutic-diagnostics for cancer. Therefore, this review will focus on discussing the important roles of miRNA expression and deregulation in the altered metabolism in cancer cells.

### MiRNAs involved in cancer cell metabolism

**Figure 1:**
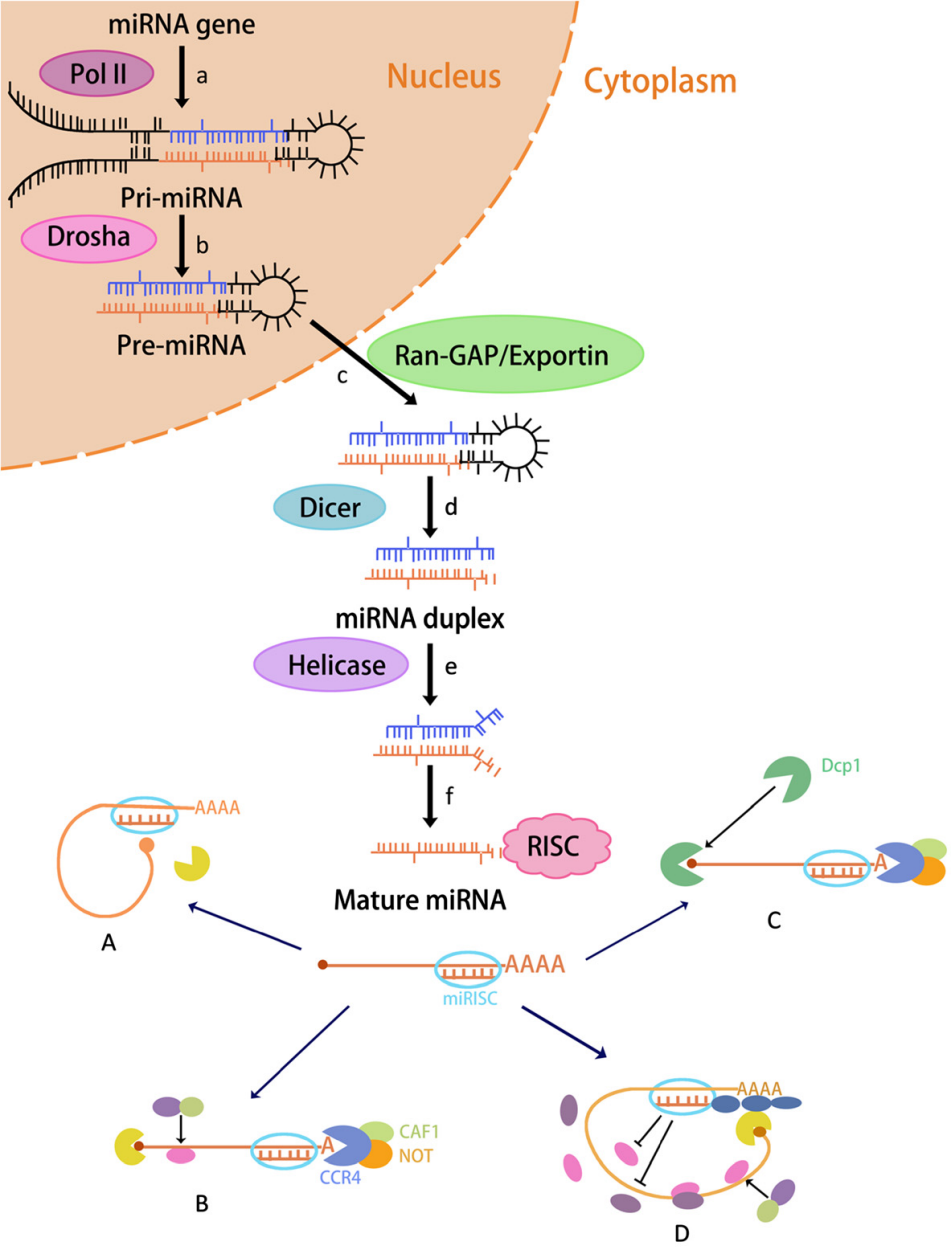
**Biological functions of miRNA. **The first step is the nuclear cleavage of the pri-miRNA, with a ~60-70 nt stem loop intermediate liberated, known as the miRNA precursor, or the pre-miRNA. Then this pre-miRNA is actively transported from the nucleus to the cytoplasm by Ran-GTP and export receptor. One end of the mature miRNA was cut by Drosha in nuclear and the other end is processed in the cytoplasm by the enzyme Dicer. Either of the strands becomes stably associated with RNA-induced silenced complex (RISC), which can be called miRISC complex. The miRISC complex inhibits the target genes by (**A**) repressing initiation at the cap recognition, (**B**) inducing deadenylation of mRNA and thereby inhibiting circularization of mRNA, (**C**) inducing ribosomes to drop off prematurely thus repressing the translation initiation and (**D**) promoting mRNA degradation.

The biogenesis of miRNAs is tightly associated with their action mechanism (Figure 1). Most miRNAs derived from independent transcription units [13,14] and are encoded by a bewildering array of genes. Their transcription is typically performed by RNA polymerase II, with transcripts capped and polyadenylated. The resulting primary or pri-miRNA transcript extends both 5’ and 3’ from the miRNA sequence. The sequential processing reaction excises the stem-loop from the remainder of the transcript to create a pre-miRNA product, which occurs in the nucleus and is mostly carried out by a nuclear member of the RNase III family (Drosha). The following step excises the terminal loop from the pre-miRNA stem to create a mature miRNA duplex of approximately 22 bp length, which is carried out by the canonical Dicer enzyme in the cytoplasm. Either of the strands becomes stably associated with RNA-induced silenced complex (RISC), which can be called miRISC complex [15,16]. The miRISC complex acts as a regulator of target gene by specially recognizing and regulating particular mRNAs to inhibit target genes [17].

A shift in glucose metabolism from oxidative phosphorylation to aerobic glycolysis was a key biochemical hallmark of tumor cells [18,19]. The altered metabolism was called “Warburg phenomenon”, which consists of an increase in glycolysis maintained in conditions of high oxygen tension and gives rise to enhanced lactate production [20,21]. Metabolic shift in cancer cells seems to be influenced by oncogene and tumor suppressor networks [22]. What’s more, most of these tumor suppressors are miRNA targets. For example, phosphatidylinositol 3-kinase, a lipid kinase that regulates the levels of phosphorylated phosphatidylinositol at the plasma membrane, plays a key role in cancer cell metabolism, which is targeted by miR-320, miR-123a, miR-422, miR-506 and miR-136.

**Figure 2:**
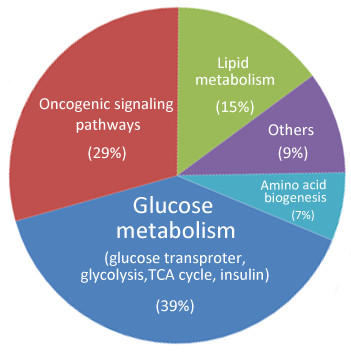
**The main miRNAs involved in metabolism of glucose, lipid and amino acid, as well as metabolism-associated oncogenic signaling pathways. **Among the total 60 miRNAs mentioned in the text, more than 20 miRNAs, including miR-375, miR-133, miR-199a, miR-138, etc., involve in glucose metabolism. And miR-14, miR-27a, miR-34a, miR-146, miR-335, miR-370, miR-122 and miR-33a/b function on lipid metabolism, including participating in controlling Acetyl-CoA and plasma cholesterol. While several miRNAs (miR-23b*, miR-29a/b, miR-277 etc.) play functions in amino acid metabolism mainly through regulating acyltransferase and α-ketoacid dehydrogenase. In addition, about 29% of the mentioned miRNAs (miR-125b, miR-504, miR-25, miR-30d, etc.) participate in metabolism-associated oncogenic signaling pathways.

There are several lines of evidence that many key molecules in cell metabolism are miRNA targets, thus giving a clue that miRNA regulates cell metabolism. Since miRNAs regulate a substantial fraction of genes in animal genomes, Tibiche and Wang systematically analyzed the human metabolic network by integrating miRNA target genes into the network [23]. They performed randomization tests to determine whether a multiple-gene-node is significantly regulated by miRNAs and defined 79 multiple-gene-nodes as miRNA targets. They merged the miRNA targets of single-gene-nodes with the multiple-gene-nodes, and found that 238 (22%) nodes are miRNA targets. The functional association analysis of miRNAs and metabolic pathways uncovered that miRNAs predominantly regulate central metabolic pathways such as amino acid biosynthesis, certain sugar and lipid metabolism (Figure 2).

### Regulation of metabolic activity by miRNAs

**Figure 3:**
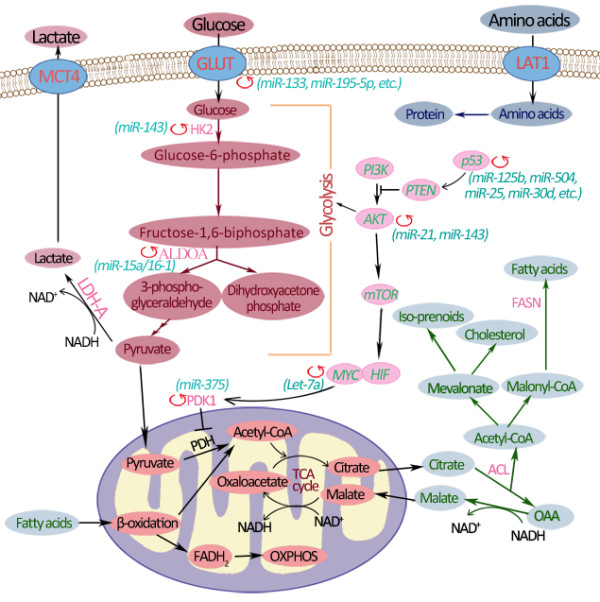
**MicroRNAs regulate cell metabolism by targeting key metabolic enzymes and multiple oncogenic signaling pathways. **MiRNAs could regulate cell metabolism by modulating the expression of metabolic transporters (like GLUT) or enzymes (HK2, ALDOA and PDK1) and acting on p53, c-Myc and AKT/mTOR signaling pathways. The steps regulated by miRNAs are indicated by red circular arrows, and the related miRNAs are listed in the bracket. FASN, fatty acid synthase; GLUT, glucose transporter; HIF, hypoxia-inducible factor; LAT1, L-type amino acid transporter 1; LDH-A, lactate dehydrogenase isoform A; MCT, monocarboxylate transporter; PDH, pyruvate dehydrogenase; PDK, pyruvate dehydrogenase kinase; PI3K, phosphatidylinositol 3-kinase.

MiRNAs regulate cell metabolic processes through complicated mechanisms, including directly targeting key molecules (transporters or enzymes / kinases) of metabolic processes and regulating multiple oncogenic signaling pathways (Figure 3). MiRNAs could directly modulate the expression of metabolic transporters or enzyme activities. In addition, MiRNAs also play pivotal roles in the expression level of transcription factors and oncogenes or tumor suppressors, including p53, c-Myc, AMPK and AKT signaling pathway.

The molecular mechanisms driving the Warburg effect in cancer cells were taken as an example to explain miRNA regulation in energy metabolism. As shown in Figure 3, several miRNAs affect gene transcription and expression of glucose transporters (GLUTs) which are responsible for transporting glucose into cytoplasm. In the initial step of glucose metabolism, glucose could be transported over a plasma membrane by GLUT3 or GLUT4 which is a target of miR-133 [24] or miR-195-5p [25]. Thus, miRNAs could directly regulate intracellular glucose levels. In the following step, the hexokinase 2 (HK2), the first rate-limiting enzyme of glycolysis, is among the top list of genes predicted and potentially regulated by multiple miRNAs including miR-143 [26]. Along the glycolysis reaction chain, fructose 1,6-bisphosphate is broken down into glyceraldehyde 3-phosphate and dihydroxyacetone phosphate, which is catalyzed by aldolase A (Aldo A) in the reversible aldol reaction. While the enzyme Aldo A is down-regulated by miR-15a/16-1cluster [27]. And the specific miRNAs in glucose metabolism will be summarized into 4 subtitles as follows, including miRNA effects on glucose uptake, glycolysis, tricarboxylic acid (TCA) cycle and insulin regulation.

On the other hand, aerobic glycolysis in tumor cells is driven by multiple miRNA-involved oncogenic signaling pathways. For example, AKT, a cardinal node in diverse signaling cascades, is regulated by miR-21 [28], which stimulates glycolysis by directly regulating glycolytic enzymes and activating downstream mammalian target of rapamycin (mTOR) activity. The regulation of several specifically signaling pathways involved in cancer cell metabolism by miRNAs will be introduced in detail in the second section of this review. Therefore, mRNA dysregulation in any step of metabolic processes contributes to metabolic abnormalities and even cancer development.

### MiRNAs regulate glucose metabolism

#### MiRNAs affect glucose uptake

GLUTs (or SLC2A) are a wide group of membrane proteins that facilitate the transport of glucose over a plasma membrane in most mammalian cells. To date, 14 members of GLUTs have been identified [29]. The amounts of the GLUT1, GLUT2, and GLUT3 transcripts were elevated in most cancer tissues, while mRNA levels of GLUT4 and GLUT5 were below sensitivity in these cancer tissues. The potential effects of the GLUTs level seem to facilitate accelerated metabolism, high glucose requirements, and increased glucose uptake in malignant cells. Several factors have been implicated in the regulation of their expressions. Hormonal, for example, ovarian hormones, particularly estrogen, could provide a mechanism of GLUT regulation [30]. In addition, hypoxic also drives GLUT expression [31] as well as metabolic-stress-induced signaling pathways, such as adenosine monophosphate-activated protein kinase (AMPK), triggering upregulation of GLUT receptors [32].

MiRNAs could regulate glucose uptake via altering the GLUTs expressions. MiR-133 has been confirmed to regulate the expression of GLUT4 by targeting KLF15 in a rat model [31]. A study in renal cell carcinoma demonstrated that down-regulated miR-199a, miR-138, miR-150 and miR-532-5p were correlated with an increased expression of GLUT-1, whereas an increased expression of miR-130b, miR-19a, miR-19b and miR-301a can result in the down-regulation of GLUT-1 [32]. MiR-195-5p has been identified as a direct regulator of GLUT3 by targeting GLUT3 3’-untranslated region in bladder cancer T24 cells [33]. Interestingly, miR-19a and miR-133a are altered in colorectal carcinoma [34], and their roles in regulating GLUT expression might explain the disordered metabolism in colorectal carcinoma. In addition, miR-130b is highly down-regulated in pancreatic tumors, and its role in regulating GLUT-1 expression might explain the increased glucose uptake in pancreatic adenocarcinoma [35].

#### Functions of miRNAs on glycolysis

Studies show that miRNAs regulate the irreversible steps in glycolysis, especially the key enzymes [33]. For example, miR-143, as an essential regulator of glycolysis, modulates glycolysis via targeting HK2 [12], which phosphorylates glucose to produce glucose 6-phosphate, thus committing glucose to the glycolytic pathway. Recently new protein targets of miRNAs have been identified by sensitive mass spectrometric studies. The oxysterol-binding-protein-related-protein 8 has been revealed as a target of miR-143 by quantitative mass spectrometry analysis [34]. For example, miR-155 could repress miR-143 thereby upregulating the expression of HK2 at the post-transcriptional level, except by activating the signal transducer and activator of transcription 3, a transcriptional activator for HK2 [35]. Besides, miR-143 inhibits the expression of HK2 both in primary keratinocytes and in head and neck squamous cell carcinoma-derived cell lines [36]. What’s more, HK2 has been validated as a miR-143 target and thus miR-143 could affect glucose metabolism in colon cancer cells [37]. Likewise, miR-143 has also been identified as an essential regulator of cancer glycolysis via targeting HK2 in human lung cancer [12]. Interestingly, the above articles were published almost at the same time. These reports all illustrated that miR-143 targets HK2 to regulate glucose metabolism in cancer cells, and it is a potential cancer therapeutic target.

Except for targeting the irreversible rate-limiting steps, miRNAs also regulate other important intermediate steps in the glycolysis pathway. The enzyme Aldo A catalyzes a reversible aldol reaction in which fructose 1,6-bisphosphate is broken down into glyceraldehyde 3-phosphate and dihydroxyacetone phosphate. In this process, miR-122 was predicted to target Aldo A [26,38], and the miR-15a/16-1 cluster could reduce the levels of Aldo A [27]. Thus miR-122 and miR-15a/16-1 cluster are involved in glycolysis in cancer cells.

#### Roles of miRNAs in TCA cycle

As described before, aerobic glycolysis in tumor cells implies conversion of glucose into pyruvate and subsequently into lactic acid [39]. Acetyl-CoA tends to be introduced into a truncated TCA cycle, with the net result that acetyl-CoA is exported into cytosol. In this truncated TCA cycle, citrate is preferentially exported to cytosol and cleaved by ATP citrate lyase (ACL) to generate oxaloacetate and acetyl-CoA. Oxaloacetate is reduced to malate, then reimported into mitochondria and reconverted to oxaloacetate in the matrix (while generating NADH that represses the TCA cycle), and it reacts with acetyl-CoA to complete the substrate cycle.

A shift in glucose metabolism from oxidative phosphorylation to aerobic glycolysis has been accepted as a common event in cancer. This process implicates different kinds of energy production pathways are mediated by diverse regulators, including miRNAs [40]. For example, miR-103 and miR-107 have been predicted in regulating acetyl CoA and lipid levels in cellular systems [41]. Additionally, a set of miRNAs, including miR-152, miR-148a, miR-148b, miR-299-5p, miR-19b, miR-122a, miR-421, miR-494 and miR-19a [23], regulate the citrate synthase gene which encodes a major enzyme in TCA cycle. Besides, miR-210, a miRNA specifically induced by HIF-1α during hypoxia, represses the iron-sulfur cluster assembly proteins (ISCU1/2) [42]. ISCU1/2 facilitates the assembly of [4Fe-4S] and [2Fe-2S] iron-sulfur clusters, which are incorporated into the TCA cycle related enzymes, like aconitase. Thereby, the effect of miR-210 on ISCU1/2 leads to decrease the activity of TCA cycle. Furthermore, miRNAs also regulate TCA cycle indirectly by acting on transcription factors Myc and HIF etc.

#### Regulation of insulin production by miRNAs

MiRNAs have shown to modulate the secretion, action and sensitivity of insulin to affect glucose uptake and production [43]. Insulin acts in concerting with glucagon to maintain glucose homeostasis. MiR-375 has been identified to be expressed selectively in pancreatic endocrine cell lines. The overexpression of miR-375 results in suppressed glucose-stimulated insulin secretion and its inhibition enhances insulin secretion [44]. Besides, *Myotrophin,* a protein implicated in exocytosis, was a validated target of miR-375. Similarly, a tantalizing new candidate target of miR-375, 3’-phosphoinositide-dependent protein kinase-1, is a key molecule in PI3-kinase signaling in pancreatic β-cells [45].

#### MiRNAs affect lipid metabolism

Several miRNAs participate in the regulation of lipid metabolism. The deletion of miR-14 increased the levels of triacylglycerol and diacylglycerol while its overexpression resulted in the converse effect, suggesting that miR-14 acts as a regulator of fat metabolism [46]. Additionally, there’s evidence that miRNAs are involved in the development and maturation of adipocytes from precursor cells called pre-adipocytes [47,48]. MiR-122 could act as an important regulator of cholesterol and fatty-acid metabolism in the adult liver [49]. The inhibition of miR-122 in normal mice resulted in reduced plasma cholesterol levels, increased hepatic fatty-acid oxidation, and a decrease in hepatic fatty-acid and cholesterol synthesis rates, but resulted in decreased plasma cholesterol levels, a significant improvement in liver steatosis and reductions in several lipogenic genes. After that, miR-122 has been found as an important regulator in liver lipid metabolism [50]. In the recent article, miR-27a has been revealed to be involved in adipocyte differentiation by binding to the PPARγ 3’-UTR, whose sequence motifs are highly conserved in mammals [51]. All these studies indicate that miRNA plays an important role in lipid metabolism.

The changes of miRNA expression in β-cell under physiopathological conditions have been illustrated before. And at least part of the detrimental effects of palmitate on pancreatic β-cells has been caused by alteration in the levels of specific miRNAs, like miR-34a and miR-146 [52]. Apart from that, the up-regulation of miR-335 has also been found in obesity by microarray analysis [53]. Besides, the expression of miR-335 was up-regulated in liver and white adipose tissue in obese mice, which was associated with an elevated body, liver and WAT weight, and hepatic triglyceride and cholesterol. Additionally, miR-370 acting via miR-122 may accumulate hepatic triglycerides by modulating initially the expression of SREBP-1c, DGAT2, and Cpt1α and, subsequently, the expression of other genes that affect lipid metabolism [54].

Recently, miR-33a/b has been discovered to govern cholesterol / lipid metabolism and energy homeostasis [55]. MiR-33a/b embeds within intron sequences of the human SREBF genes and controls the levels of ATP-binding cassette transporter ABCA1, a cholesterol efflux pump critical for high-density lipoprotein synthesis and reversing cholesterol transport from peripheral tissues [56,57]. MiR-33a/b also acts in the lipid homeostasis pathway by controlling the expression of fatty acid β-oxidation genes including carnitine *O*-octanoyltransferase, hydroxyacyl-CoA-dehydrogenase, and carnitine palmitoyltransferase 1A, as well as energy homeostasis regulators AMPK a1, SIRT6, and insulin receptor substrate 2 [58]. These reports bring us a further view of miRNA function on lipid metabolism.

#### Effects of miRNA in amino acid metabolism

Amino acid metabolism is linked to biosynthesis of protein, nucleotide and lipids, redox homeostasis, and energy metabolism. MiR-23b* (expressed from the 3'-arm) mediates proline oxidase, the first enzyme in proline catabolism, down-regulation in human kidney tumors [59]. Furthermore, the metabolic link between proline and glutamine afforded by Myc emphasizes the complexity of tumor metabolism. While miR-122 was reported to downregulate the high affinity cationic amino acid transporter CAT-1 [60], thereby regulating amino acid metabolism. Involving evidences have been found in *Drosophila*, where miR-277 plays a role as a metabolic switch controlling amino acid catabolism by bioinformatics approaches [61]. Additionally, miR29b has been identified to control the component of the branched chain a-ketoacid dehydrogenase complex, which catalyzes the irreversible step in branched chain amino acids (including leucine, isoleucine and valine) catabolism [62], suggesting that miR-29b exerts effects of controlling on amino acid catabolism.

#### miRNA regulation of signaling pathways in cell metabolism

The intertwined connections between aberrant expression of microRNAs and unbalanced signaling pathways contribute to abnormal cell metabolism and carcinogenesis. The specific p53, c-Myc, AMPK and AKT signaling pathways are included to clarify their roles in miRNA-mediated metabolism.

#### p53 pathway

p53, one of the most common tumour suppressor genes, functions to prevent tumour development by inhibiting the outgrowth of stressed or damaged cells. In addition to well established functions to block cell proliferation, recent studies have revealed regulation roles for p53 involved in metabolism [63]. The p53 can inhibit the expression of GLUT-1, GLUT-4, phosphoglyceromutase and TIGAR to affect glycolysis. TIGAR is TP53-induced glycolysis and apoptosis regulator protein, and it inhibits the glycolytic enzyme PFKFB2 [64]. Additonally, p53 could also activate the expression of synthesis of cytochrome c oxidase 2 at transcriptional level [65] and induce the expression of the ribonucleotide reductase subunit p53R2 [66], leading to the restraint on glycolytic rate.

Several miRNAs are able to control p53 activity. The miR- 125b has been identified as a negative regulator of p53 in both zebrafish and human [67]. To date, miRNAs including miR-125b, miR-504, miR-25, miR-30d, miR-34a, miR-122, miR-29, miR-192, miR-194 and miR-215 have been shown to regulate p53 abundance and/or activity. Among these, miR-125b, miR-504, miR-25 and miR-30d negatively regulate p53 by binding to its 3'UTR whereas the others indirectly influence p53 abundance and/or activity by regulating the regulators of p53 [68]. The functions of these miRNAs on p53 give a clue of their effects in cancer cell metabolism.

#### c-Myc pathway

The c-Myc is a transcription factor that regulates the expression of genes involved in nucleotide metabolism, DNA replication, and ribosomal and mitochondrial biogenesis. Studies in the past few years have led to the identification of miRNAs as novel regulators of c-Myc activity. A mutated version of Myc leads to the unregulated expression of many genes, some of which are involved in cell proliferation and results in the formation of cancer [69]. For example, c-Myc has crucial roles in glutamine metabolism mediated by miR-23b [70]. Moreover, in concerts with HIF1 to regulate glucose uptake and glycolytic enzyme expression, thus favouring tumour growth in hostile environments [71].

The regulation of Myc mRNA by let-7a has been confirmed [72]. Similiarly, the overexpression of let-7a can inhibit the growth of lung cancer transplanted subcutaneously in nude mice by suppression of k-Ras and c-Myc [73]. Inspiringly, c-Myc transcriptionally represses miR-23a and miR-23b, resulting in increased expression of mitochondrial glutaminase, enhancing glutamine catabolism through increased mitochondrial glutaminase expression [6].

#### AMPK pathway

AMPK acts as a metabolic master switch regulating several intracellular systems including the cellular uptake of glucose, the β-oxidation of fatty acids and the biogenesis of GLUT4 and mitochondria [74]. AMPK controls glucose homeostasis by regulating metabolism in multiple peripheral tissues, such as skeletal muscle, liver, adipose tissues, and pancreatic β cells [75].

The functions of miR-375 on glucose homeostasis have been studied [76]. Total 381 putative direct targets of miR-375 were selected, which contained a miR-375 recognition motif, and confirmed 10 of these genes, involving caveolin1 [77,78], inhibitor of DNA binding 3 [79,80], *Smarca2*, Ras-dexamethasone-induced-1 [81], regulator of G protein signaling 16 [82], eukaryotic elongation factor 1 epsilon 1, apoptosis-inducing factor, mitochondrion-associated 1, cell adhesion molecule 1, HuD antigen [83], and complement component 1 q subcomponent binding protein. Published data have shown that some of these genes play a role in AMPK signaling, inducing apoptosis and inhibiting normal developmental growth processes or the proliferation of tumors.

#### AKT pathway

The PI3K/AKT/mTOR pathway is an intracellular signalling pathway, which is important in apoptosis. It has risen to prominence as a key regulator of cell cycle proliferation, growth, survival, protein synthesis, and glucose metabolism [84]. Activation of PI3K leads to the activation of downstream effectors including Akt and mTOR that support cellular biosynthesis [85-87]. Enhanced PI3K/Akt signal increases the expression of nutrient transporters, enabling increased uptake of glucose, amino acids, and other nutrients. Additionally, Akt-dependent stimulation of hexokinase and phosphofructokinase drives glycolysis. Furthermore, AKT-involved signaling enhances transcription of genes to involve in glycolysis and lipid genesis [88-90].

Regulation of this pathway by miRNAs mainly results in altered glucose and lipid metabolism. For example, miR-21, which inhibits a negative regulator PTEN of the PIK/AKT pathway, is induced in gemcitabine-resistant pancreatic cancer cells [28]. And AKT pathway can involve in glycolysis by directly regulating glycolytic enzymes and activating downstream mTOR activity. Similiarly, ORP8 has been identied as an miR-143 target and the reduction of ORP8 expression in cultured liver cells impairs the ability of insulin to induce AKT activation, revealing an ORP8-dependent mechanism of AKT regulation [91].

#### MiRNAs affect multiple targets in regulatory networks

Certain miRNAs have also been shown to affect multiple targets in linear pathways or interconnected nodes in regulatory networks [25], thereby exerting a larger cumulative effect [9,55]. For example, miR-33a and miR-33b, as described before, interact with the SREBP transcription factors to regulate cholesterol and lipid homeostasis. Furthermore, they may also influence insulin signaling and glucose regulation by targeting IRS2, SIRT6 and AMPK α1 [58]. MiR-34a, a miRNA that may have important function in a network with SIRT1 and p53, has additionally been implicated in cholesterol, lipid and energy homeostasis [52,68]. MiRNAs typically have rather modest effects on target protein levels, and combinatorial actions on multiple functionally related targets are probably required for single miRNAs to significantly influence a complex biological process such as metabolic homeostasis.

#### MiRNAs as biomarkers for human cancer

By targeting and controlling the expression of mRNA, miRNAs can control highly complex signal transduction pathways and multiple metabolic processes, which are usually involved in different oncogenic pathways [92]. The knowledge that miRNA expression is frequently dysregulated in cancer has uncovered an entirely new repertoire of molecular factors upstream of gene expression, with exciting potential as novel biomarkers and therapeutic targets in cancer [93]. Exploiting the unique characteristics of these molecules including their stability, tissue specificity, ease of detection and manipulation, will bring clinicians ever closer to achieving the goal of individualized cancer treatment [94].

On the one hand, miRNAs are produced in a tissue-specific manner, and changes in miRNA within a tissue type can be correlated with disease status. The tissue concentrations of specific miRNAs have been associated with tumor invasiveness, metastatic potential, and other clinical characteristics for several types of cancers, including chronic lymphocytic leukemia, and breast, colorectal, hepatic, lung, pancreatic, and prostate cancers [95]. On the other hand, there has been an accumulating body of evidence to support circulating miRNAs as non-invasive, sensitive biomarkers of disease states, particularly cancers (breast, lung, pancreas, ovarian, and prostate) [96]. For example, miR-9 and miR-9* (expressed from the 3' mature sequence), mostly neuronal and thus expressed in central nervous system tumors but absent in other tumors, present their potential as tumor markers [97]. In addition, the reduced levels of miR-126, members of the miR-17-92 cluster, inflammation-related miR-155, and smooth muscle-enriched miR-145 in patients with coronary artery disease compared with healthy controls [98]. What’s more, published data showed that plasma miR-29a and miR-92a have strong potential as novel noninvasive biomarkers for early detection of colorectal carcinoma [99].

Furthermore, since they are abundant in blood, easy to measure, highly stable and disease associated, serum microRNAs are attractive disease biomarkers [100]. There have been over 200 publications on circulating miRNA in cancers including prostate, breast, colon, lung, ovarian and leukemia since 2008. Considering the sources of variation, state of microRNA in plasma and origin and implications for disease specificity, miRNA expression profiles of potential patients could be assessed by measuring circulating miRNAs in patient serum. This profile could be hopefully used for early detection of cancer.

## Conclusion and perspective

**Figure 4:**
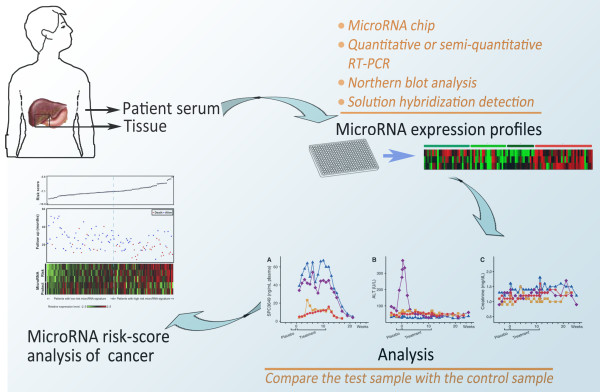
**MiRNA-based diagnosis strategies for cancer. **The workflow of detecting cancer based on miRNA profiling is included sample (serum or tissue) collection, miRNA expression profiling, data analysis of miRNA expression level and cancer risk assessment. The level of the at least one miRNA gene product can be measured using a variety of techniques (microRNA chip, quantitative or semi-quantitative RT-PCR, northern blot analysis, solution hybridization detection etc.) to provide a profile for the test sample. The level of at least one miRNA gene product in a test sample from the subject is compared to that in a control sample. A significantly increased or decreased alteration in the level of the miRNA gene product in the detected sample is indicative of the subject either having or being at risk for developing a cancer.

MiRNAs are important regulators of numerous aspects of metabolic homeostasis, physiology and disease. In general, miRNAs could mainly have two ways to regulate cellular metabolism. MiRNAs could regulate transcription factors or signaling proteins, which in turn regulate metabolic enzymes. Alternatively, miRNAs could regulate the production of certain metabolites by directly regulating the genes that encode metabolic enzymes [101]. In addition, miRNAs could regulate mRNAs through chromatin remodeling [102]. The emergence of miRNAs as important regulators of metabolism has garnered much interest not only from a scientific point of view but also from a clinical perspective. The function of miRNAs on cellular metabolism reveals molecular strategies for controlling metabolic flux by miRNAs in living organisms, thus lighting up one aspect of miRNA therapeutics. MiRNAs are promising in the diagnosis of cancer, drug target identification and clinical treatment in the future (Figure 4). The use of miRNAs, such as oligonucleotide complementary [103] or antisense oligonucleotides [104] in miRNA inhibition, to suppress cell metabolism altering will hopefully lead to a new therapeutic strategy for malignant cancer [105,106]. For example, endothelial miR-126 is deregulated in patients with type 2 diabetes, which may ultimately lead to novel biomarkers for risk estimation and classification and could be exploited for miRNA-based therapeutic interventions of vascular complications associated with this disease [107].

So far, a variety of new strategies to identify and characterize the targets of individual miRNAs have been developed. Because miRNAs can also regulate other non-coding RNAs, these interactions will increase the complexity of gene regulation. Moreover, cost-effective miRNA profiling strategies and larger studies are needed to determine its advantage for cancer diagnosis. Additionally, a new class of miRNA-based drugs that are capable of targeting molecules outside the range of traditional medicinal chemistry, their clinical implementation will require improvements in drug composition and delivery. Since these challenges lie on the way, molecular strategies for cancer therapy by miRNAs are still in their infancy. Nevertheless, the successful development of miRNA biology technologies could ultimately translate our understanding of miRNA functions in cancer into strategies for the control of cancer.

## Abbreviations

ACL: ATP citrate lyase; AldoA: Aldolase A; AMPK: Adenosine monophosphate-activated protein kinase; GLUT: Glucose transporter; ISCU1/2: Iron-sulfur cluster assembly proteins; HIF1: Hypoxia-inducible factor 1; HK2: Hexokinase 2; MiRNA: MicroRNA; MTOR: Mammalian target of rapamycin; ORP: Oxysterol-binding-protein-related-protein; RISC: RNA-induced silenced complex; TCA: Tricarboxylic acid.

## Competing interests

The authors declare that they have no competing interests.

## Authors’ contributions

All authors participated in the preparation of the manuscript, read and approved the final manuscript.
